# User-provider experiences of the implementation of KidzAlive-driven child-friendly spaces in KwaZulu-Natal, South Africa

**DOI:** 10.1186/s12889-019-7712-2

**Published:** 2020-01-21

**Authors:** Chipo Mutambo, Kemist Shumba, Khumbulani W. Hlongwana

**Affiliations:** 1 The Discipline of Public Health Medicine, School of Nursing and Public Health, University of KwaZulu-Natal, Howard College Campus, King George Avenue, Durban, 4041 South Africa; 2 The Discipline of Psychology, School of Applied Human Sciences, University of KwaZulu-Natal, Office IX06 TB Davies Ext, Howard College Campus, Durban, 4041 South Africa; 3 School of Nursing & Public Health, College of Health Sciences, University of KwaZulu-Natal, Howard College Campus, King George Avenue, Durban, 4041 South Africa; 4 Department of Public Health Medicine, University of KwaZulu-Natal, George Campbell Building, Room 217, 2nd Floor, King George V Avenue, Glenwood, Durban, KwaZulu-Natal 4041 South Africa

**Keywords:** Child-friendly spaces, Child-centred care, Children, Primary caregivers, Play therapy, Child-friendly environment, HIV, KidzAlive trained and mentored HCWs

## Abstract

**Background:**

KidzAlive is a child-centred intervention aimed at improving the quality of HIV care for children in South Africa. Through this intervention, 10 child-friendly spaces were created in 10 primary healthcare centres (PHCs) in KwaZulu-Natal to enhance child-centred HIV care. However, the user-provider experiences of these child-friendly spaces in these facilities have not been explored. This paper addresses this gap.

**Methods:**

We conducted qualitative interviews with children (*n* = 30), their primary caregivers (PCGs) (*n* = 30), and KidzAlive trained healthcare workers (HCWs) (*n* = 20) using and providing child-friendly spaces, respectively. Data were generated, using a semi-structured interview guide printed in both English and IsiZulu. The interviews were audio-recorded transcribed and translated to English by a research team member competent in both languages. Data were imported to NVivo 10 for thematic analysis. The COREQ checklist was used to ensure that the study adheres to quality standards for reporting qualitative research.

**Results:**

Child-friendly spaces contributed to the centredness of care for children in PHCs. This was evidenced by the increased involvement and participation of children, increased PCGs’ participation in the care of their children and a positive transformation of the PHC to a therapeutic environment for children. Several barriers impeding the success of child-friendly spaces were reported including space challenges; clashing health facility priorities; inadequate management support; inadequate training on how to maximise the child-friendly spaces and lastly the inappropriateness of existing child-friendly spaces for much older children.

**Conclusion:**

Child-friendly spaces promote HIV positive children’s right to participation and agency in accessing care. However, more rigorous quantitative evaluation is required to determine their impact on children’s HIV-related health outcomes.

## Key points

- Child-friendly spaces were enacted in PHCs in KwaZulu-Natal to create a healing environment for children living with HIV as part of a child-centred care initiative called KidzAlive.
- We interviewed users and providers of these child-friendly spaces to explore their experiences in these spaces.
- Users reported that child-friendly spaces increased their involvement and participation; increased PCGs’ participation in the care of their children and positively transformed the PHC facility into a therapeutic environment.
- Several barriers impeding the success of child-friendly spaces were reported including space constraints; clashing health facility priorities; inadequate management support; inadequate training on how to maximise the child-friendly spaces and their inappropriateness for older children.

## Background

Children have, for some time, lagged behind in the global response to HIV [1, 2]. However, the recent increase in advocacy for child-centred approaches in HIV programmes is anticipated to mitigate this problem through being responsive to the needs of HIV seropositive children [2–6]. This is fuelled by the global paradigm shift from disease-focused care and healthcare worker (HCW) paternalism to patient-centred care [7]. The Institute of Medicine (IOM) defines patient-centred care as: “Providing care that is respectful of, and responsive to, individual patient preferences, needs and values, and ensuring that patient values guide all clinical decisions” [8]. Child-centred care is built on the principle of patient-centred care [6, 9]. The United Nations Convention on the Rights of the Child (UNCRC) serves as the *Magna Carta* of the child-rights based approach in promoting the provision of child-centred healthcare [6, 10]. It integrates children’s rights, parents’ rights and contemporary child protection policies [9–11]. However, studies also synonymise child-centred care with family-centred care [6, 12]. The family-centred care philosophy posits that the family is the unit of care and that healthcare for children is a joint effort between PCGs, other family members and the HCWs who are given the responsibility of ensuring that the child is provided with care [10]. Although both concepts are cut from the same cloth of “centredness”, family-centred care is widely celebrated and ingrained in various healthcare policies and guidelines [12], yet child-centred care, is not, and remains largely elusive. However, recent publications have criticised family-centred care approaches for perpetuating HCW paternalistic ideologies and PCG dominance, thus creating an asymmetrical relationship between the child, HCW and PCG, which stifles children’s right to participation and decision-making in accessing healthcare service [12–14].

In support of child-centred care, some studies applaud this approach for being sensitive to the needs of each child [10, 15]. Accordingly, it addresses some of the child-related complexities that may have been overlooked by the generalised patient-centred care approach [16]. Similar to patient-centred care, key principles underpinning the concept of holism are applicable to child-centred care, which include providing healthcare that puts the child at the forefront of care [6, 9]; amplification of the voice of the child [9, 17]; prioritisation of the child’s needs and values [18]; listening to the child’s experiences and interests [9, 10]; and providing age-friendly information to increase children’s understanding of their illness and how to manage it [11, 15]. Other scholars suggest that child-centred care approaches celebrate childhood and acknowledge the cognitive, legal and cultural challenges and limitations associated with this stage of human development [9, 10]. In addressing these gaps, child-centred approaches recognise children as “agentic beings” with the ability to actively influence their healthcare [6, 19].

However, the adoption and application of child-centred care approaches to the delivery of HIV services is still very limited. Nevertheless, there is evidence of the application of some of its constructs to HIV programmes for children [20–22]. Furthermore, the concept has still not been defined extensively especially in the context of HIV, although some scholars are gradually realising the value of tailoring HIV services to be in sync with the needs of the child [11, 12, 14, 22, 23]. A good example of such an intervention is the promotion of status disclosure for children living with HIV conducted in Namibia where a cartoon-based storybook was used to facilitate disclosure [24, 25]. By applying the right to both information and participation, healthcare providers are providing children with age-appropriate information to facilitate disclosure and potentially improve medication adherence and achieve positive health outcomes [26–28]. The sharing of information through consultative sessions between HIV seropositive children, HCWs and PCGs has been shown to forge partnerships between the parties involved, and promote transparency and truthfulness, which are key to promoting status disclosure [26–28].

Child-centred care approaches do not only prioritise the child’s needs but involve the key players that include the child’s PCG(s), family, the broader community and the healthcare facility [6, 10, 29]. One of the key components of child-centred HIV care is the creation of a child-friendly healthcare environment [10, 30]. There is evidence suggesting that modifying the healthcare environment for children using visual art and spaces where children can play with toys inside a healthcare setting can marginally improve both their healthcare experiences and care satisfaction [31, 32]. Studies have attributed the poor health-seeking behaviours of both children and their PCGs to the unwelcoming and dreary PHC facility environment [33, 34], compounded with an overpopulated healthcare facility with anxious patients, rushing HCWs, and overwhelming clinical equipment and looming tests [33–35].

To reduce anxiety among children visiting health facilities and to improve their experiences of healthcare, the concept of child-friendly spaces has been suggested. Similar to the concept of child-centred care, child-friendly spaces are a relatively new concept. It was coined by humanitarian child protection agencies in 2008 who defined it as a “space that supports the resilience and well-being of children and young people who have experienced disasters through community organised, structured activities conducted in a safe, child-friendly, and stimulating environment” [36]. The concept of child-friendly spaces (also referred to as “safe spaces”, “child-centred spaces” and “child protection centres” focusses on the children’s need for a protected environment, to learn, express themselves, build self-esteem, socialise and play – all of which are components of healthy psychosocial development [37]. These spaces play a pivotal role in easing difficult transitions or providing a stable environment for children during difficult or traumatic periods [38]. They also provide a contact point where HCWs and other professionals can assist vulnerable children. The concept encompasses not only the physical space but the associated programmes that are delivered to children and their PCGs [39]. Initially developed to support children during times of humanitarian crises, many international organisations, including the United Nations Children’s Fund (UNICEF) and Save the Children, have adopted the child-friendly space, as a key intervention for protecting children at risk of harm in high conflict countries [36, 40].

Some broad guidelines have been developed to guide the development of a child-friendly space [38]. These guidelines suggest that a child-friendly space should uphold the provisions made in the UNCRC (1989) treaty that include physical safety; designed and operated in a participatory and culturally appropriate manner; local community involvement; highly inclusive and non-discriminatory; ability to incorporate a wide range of appropriate activities; and able to employ sensitive and well-trained staff [40]. However, child-friendly spaces have mostly been praised for their responsiveness and sensitivity to the needs of children in crisis, yet very little is documented regarding their pitfalls.

The child-friendly spaces model is inherently adaptable to a variety of contexts and can be modified for different settings and age groups. However, there is currently a lack of data that rigorously evaluate the efficacy of child-friendly spaces. The existing data mainly focusses on the impact of child-friendly spaces during humanitarian crisis situations, such as emergency and disaster [37]. An evaluation of ‘child-centred spaces’ in Northern Uganda found that these spaces had a tangible benefit for children, translating into children who experienced less emotional distress, displaying fewer behavioural issues and better social skills. The ‘child-centred spaces’ programme also resulted in children having improved knowledge about hygiene, communication skills, literacy and numeracy [41]. The child-friendly space also served as an information hub where children acquired knowledge that could then be disseminated to their community and peers. Other studies reported that access to a child-friendly spaces helps decrease the sexual exploitation and abuse of children [42], relieves anxiety, and withdrawal and improves interactions between children and parents or PCGs [43].

The value of using child-friendly spaces to assist children who are vulnerable to health-related crises, such as the HIV epidemic is gaining traction in South Africa [44]. Children grapple with such issues as understanding what HIV is, and how it affects them [45], disclosure of their own or a caregiver’s HIV seropositive status [46], adherence to lifelong antiretroviral therapy (ART) regimens and a lack of entry points into the healthcare system for HIV testing, often perceiving adult HIV clinics as frightening and unwelcoming [47]. Therefore, child-friendly spaces now serve as enabling environments where children receive HIV services in a familiar environment which uses play to ensure their engagement, to fight stigma and build their resilience. Child-friendly spaces are expected to help reduce the fear, anxiety and distress often associated with HIV clinic visits, thereby increasing treatment adherence and the willingness to attend scheduled appointments.

Recognising the huge burden of children living with HIV in South Africa and the fact that these children spend a substantial amount of time at healthcare centres, Zoë-life,  a local non-governmental organisation (NGO) introduced child-friendly spaces in 2008 through its KidzAlive intervention as a way of creating an enabling environment for child-involvement in HIV care and treatment programmes. Despite the increasing demand for KidzAlive and its child-friendly spaces in South Africa, the intervention has not been evaluated. Apart from the informal feedback provided by KidzAlive trainers and some trainees over the years, little is known about its impact on the overall quality of care, health experiences and health outcomes of children receiving HIV services in PHC facilities in KwaZulu-Natal, South Africa. Furthermore, KidzAlive is a good example of an intervention which has been taken to scale by the government without an evidence basis to support its effectiveness, acceptability and utility. Formalising this knowledge is worthwhile, especially in the wake of limited resources and acceleration of effective and innovative change ideas for HIV care for children. Given this paucity of research on the impact of KidzAlive in general, this study partly closes some of the research gaps related to child-friendly spaces which are a key component KidzAlive; by exploring the unique experiences of children, their PCGs and HCWs. To our knowledge, no such study has been conducted, at least in South African PHC settings. Findings from this study will provide insight to Zoë-life, the developers of the intervention, their funders and the South African Department of Health regarding the intervention’s acceptability and utility. This knowledge can be used to motivate for further commitment to scale-up the intervention or abandon it.

## Methods

### Program overview

Recognising the huge burden of children living with HIV in South Africa and the fact that these children spend a substantial amount of time at healthcare centres, Zoë-life, a local NGO, introduced the KidzAlive intervention in 2006. KidzAlive is a unique child-centred HIV care and support model which is driven by frontline healthcare workers proving HIV care to children aged 2–12 years and their PCGs in rural and urban PHC settings and local community organisations in South Africa (www.kidzalive.co.za). Zoë-life describes KidzAlive as an integrated child-centred package of care that offers a set of psychosocial and wellness services to children aged 2–12 years living with HIV and AIDS. The age-sensitive content of KidzAlive is informed by child-development theories including Bandura’s Social Learning Theory [48], Piaget’s Cognitive Developmental Theory [49] and Vygotsky’s Sociocultural Theory [50, 51]. In addition, these theories are operationalised in KidzAlive using the Play Therapy Theory [52–54]. The Association of Play Therapy (APT) has defined play therapy as: “The systematic use of a theoretical model to establish an interpersonal process wherein a trained play therapist uses the therapeutic powers of play to help clients prevent or resolve psychosocial difficulties and achieve optimal growth and development.” [55].

To scale-up the KidzAlive intervention, Zoë-life received funding from the United States President’s Emergency Plan for AIDS Relief (PEPFAR), a United States government’s initiative to help save the lives of those infected with, and affected by HIV/AIDS around the world [56] in 2010 to print capacity building resources and provide training and mentorship to HCWs providing HIV services to children between 2008 and 2010. In 2014, Zoë-life received additional funding from PEPFAR; to scale-up the KidzAlive intervention in eleven (11) PEPFAR Priority Districts in South Africa. During this time, the National Department of Health (NDoH) requested Zoë-life to contribute to the development of the differentiated care service delivery interventions by including KidzAlive as the differentiated care model for children. This collaborative effort by Zoë-life and the NDoH resulted in the incorporation of child-centred care approaches in the current Adherence Guidelines [57] and National Disclosure Guidelines for children and adolescents [58]. As a result of this work, the KidzAlive child-centred care approach has been indorsed by various provincial health departments including KwaZulu-Natal as their differentiated HIV care model for all children. The KwaZulu-Natal Provincial Department of Health receives technical support from Zoë-life to mainstream the KidzAlive child-centred approach into HIV service delivery for children in PHCs across four districts (uMgungundlovu, eThekwini, uMkhanyakude and Zululand) in this province. To date, more than 400 HCWs in KwaZulu-Natal have been KidzAlive trained, mentored and provided with KidzAlive job aids while 80 have received the child-friendly spaces toolboxes to create child-friendly spaces.

### Components of the KidzAlive intervention

KidzAlive has three components, which are the KidzAlive HCW training and mentorship intervention (capacity building), KidzAlive talk tool storybook (talk tool), and child-friendly spaces. These are described in detail as follows:

#### KidzAlive HCW training and mentorship

To select HCWs to participate in the KidzAlive capacity building programme, Zoë-life is guided by the Department of Health’s district management teams, PHC managers and the Department of Health’s regional training centre to nominate two HCWs (nurse and HIV counsellor) per facility that are already providing HIV services to children including HIV testing and counselling, disclosure counselling, ART initiation, adherence counselling. A standard KidzAlive capacity building process involves the delivery of a deductive five-day classroom training followed by a one-month-long on-site one-on-one mentorship experience for each KidzAlive trained HCW. This training and mentorship process is conducted by Zoë-life’s KidzAlive trainers who are nurses and social workers with advanced qualifications and experience in HIV care and support of children. Each KidzAlive trainer is responsible for mentoring all the HCWs that they would have trained. This capacity building process serves as a vehicle for introducing certified child-friendly service providers to deliver age-appropriate HIV services to children in PHC facilities. In general, the training covers the following but is not limited to: child-rights, play therapy techniques, communicating with children and their PCGs, creating and utilising child-friendly spaces in HIV care, stages of childhood development, how to deal with children in distress and how to utilise story telling techniques to communicate with children.

Following the training, the KidzAlive trained HCWs are then mentored by the same person who trained them, referred to as a KidzAlive mentor. The mentorship process takes place at the PHC facility or community-based organisation where the KidzAlive trained HCWs usually provide care to children. The mentor initiates the mentorship process by demonstrating the provision of HIV services using three (3) cases (HIV Testing, HIV Disclosure, and Adherence Counselling), which is termed preceptorship. Following preceptorship, the KidzAlive trained HCW is then asked to provide child-friendly care to five (5) cases of child-PCG pairs under the guidance and observation of the mentor. During this process, the KidzAlive mentor uses a KidzAlive mentorship checklist to assess the mentee’s competence in specific child-friendly skills. The HCW is deemed competent if the mentor is satisfied that the mentee has adequately demonstrated mastery of the key competencies for each skill taught during the training and demonstrated during preceptorship.

#### KidzAlive talk tool storybook (talk tool)

KidzAlive trained HCWs are provided with the talk tool, which can be described as a colourful cartoon-based health education tool which is used by HCWs when providing HIV services to children. It is characterised by catchy illustrations that tell the story of *Sibusiso the Frog*. The story’s three protagonists are “*Sibusiso the Frog*”, his grandfather “*Mkhulu Noah*” and one “*Nurse Thelma*”. In the story, *Sibusiso* learns about HIV, coping with stigma, and the importance of adherence to treatment. Both “*Mkhulu Noah*” and “*Nurse Thelma*” provide the necessary social support structure to *Sibusiso* in managing his HIV status. This story uses age-appropriate language, for example; referring to medication as “Goodnight medicine that puts germs to sleep”, HIV as a “Germ in your body” and CD4 cells as “Soldiers that fight the germs”. During KidzAlive training, HCWs are taught simple terminology to use when interacting with children of different ages and disclosure status. The storybook is innovatively designed, such that it has a story side facing the KidzAlive trained HCW (narrative text of the story) while the opposite side facing the child shows a colourful cartoon illustration of the story being narrated by the HCW. When providing HIV care to the child in the child-friendly space, the KidzAlive trained HCW sits opposite the child and positions the talk tool on a flat surface (usually a table).

#### Child-friendly spaces

During KidzAlive training, HCWs are trained on how to create child-friendly spaces for children and how to fully utilise these spaces during service delivery. Following the KidzAlive training, HCWs are provided with a child-friendly space toolbox with equipment for creating a simple child-friendly space. Child-friendly spaces vary in size but in general they include two colourful children’s chairs, a small table, toys, colour-in templates with characters from the talk tool. Children can decorate the space using their artwork crafted during consultation sessions. In these spaces, KidzAlive trained HCWs use their newly acquired play therapy skills to engage the children during healthcare consultations and provide them with positive healthcare experiences every time they seek care at PHC facilities (https://www.youtube.com/watch?v=LN0pbLsMgJk).

### Study design

We adopted a qualitative explorative, descriptive and contextual design, rooted in interpretive methodology [59] to explore the experiences of HCWs, PCGs and HIV seropositive children on the use of child-friendly spaces in PHC facilities in KwaZulu-Natal. We sought to elicit rich data that can be useful to the child-friendly spaces intervention. The COREQ checklist [60] was used to ensure that the study adheres to quality standards for reporting qualitative research.

In September 2018, a convenience sample of HCWs from 40 PHC facilities across four districts [eThekwini (urban), uMkhanyakude (rural), Zululand (rural) and uMgungundlovu (urban)] were trained, mentored and certified as KidzAlive trained HCWs under the KidzAlive programme as part of a broader evaluation study. The programme implementation period was September 2018 to February 2019. It included technical support by Zoë-life to create child-friendly spaces at the HCWs’ workplaces and using them to provide HIV services to children aged 5–12 years and their PCGs. During the implementation period, we conducted face-to-face in-depth interviews with 30 HIV seropositive children, 30 PCGs and 20 KidzAlive trained HCWs using three different semi-structured interview guides for each sample. These participants were selected purposively on the basis of having participated in the programme and their being information rich.

### Training of research assistants and pretesting data collection instruments

Cognisant that the researcher is the key instrument in qualitative research [61], four research assistants (RAs) experienced in qualitative research methods, were recruited and given additional/refresher training on qualitative data generation, prior to commencing the fieldwork. These RAs were fluent in both English and isiZulu (the predominant language in KwaZulu-Natal) to enable translations and back-translations of all study materials, as required. Apart from their prior qualitative research experience, the RAs were HCWs whose scope of work involved HIV counselling, testing, and providing disclosure and adherence support. The interviews were conducted in the language most preferred by the participants, which was either isiZulu or English. The IsiZulu version of the interview guide was translated to English by a certified and experienced language translator. Interview questions included participants’ perceptions of the child-friendly space, and how it had impacted the healthcare service they had received/given during their PHC visits. The data collection instruments were pretested in King Cetshwayo District, KwaZulu-Natal on a sample of six HCWs and 10 child-PCG pairs who had also received KidzAlive training and had enacted child-friendly spaces respectively.

### Data generation process

#### Interviews with children and their PCGs

RAs visited PHC facilities with child-friendly spaces to introduce the study to eligible child-PCG pairs. They informed the eligible child-PCG pairs about the study and sought PCG consent, and parental consent allowing us to interview their children. Assent to participate was sought from the children. A child-friendly story-based information sheet and assent form were developed to improve children’s understanding of the research process. Thereafter, the child-PCG pairs were referred to the HIV counselling room with a child-friendly space where they were provided with the HIV service by the KidzAlive trained HCWs with the aid of the talk tool. After receiving the service, the child-PCG pair were referred to another private room where they were each interviewed by the RA for about an hour.

#### Interviews with KidzAlive trained HCWs

KidzAlive trained HCWs were interviewed for about an hour at the end of the initial 6 months’ post-training and mentorship period. They signed informed consent prior to each interview.

Data saturation for HCWs and child-PCG pairs was reached at 20 interviews and 30 interviews respectively. There were no refusals during the data collection process. Due to funding constraints and consideration of the ease of accessibility of child-PCG pairs. Repeat interviews were not conducted. However, we audio-recorded all interviews and took hand-written field notes during each interview with permission from the participants.

### Data analysis

The data analysis process started with transcription and translation of the data from IsiZulu to English which was done by a qualified and experienced bilingual translator with knowledge of the KidzAlive intervention to ensure conceptual equivalence [62]. Following the translation process, transcription was done by two researchers (CM and KS) to ensure rigour. Due to the fact that we were addressing specific research questions, we conducted a theoretical thematic analysis. Thematic data analysis was iteratively conducted manually and electronically by KS and CM, using the five stages of Ritchie and Spencer’s [17] data analysis framework:

1. Familiarisation with the data through reading all the transcripts and listening to the audio recordings.
2. Generation of initial codes using an *open coding* method where we only coded each segment of data that was relevant to or captured something interesting about our research question. We did not code every piece of text. We sifted through the hardcopies of the transcripts manually using highlighters to improve the efficiency of the process and to immerse ourselves into the data.
3. Development of a thematic framework drawing themes from the coded data.
4. Application of the thematic framework to all the data, using NVivo (Version 10) qualitative data analysis software.
5. Charting of the data, enabling systematic comparisons between data sets.
6. Analysis of the charts for patterns and associations between and within each unit of analysis. Codes were clustered to form sub-themes that captured the aggregated contributions. These sub-themes led to the development of themes that adequately captured the contours of the coded data.

### Trustworthiness of the findings

The trustworthiness of study findings was ensured through credibility, dependability, transferability, and confirmability [18]. To ensure credibility, we used a purposive sample of the users of child-friendly spaces and paid attention to negative cases during analysis. Furthermore, we retrieved and presented verbatim quotes from audio recordings and performed analyst triangulation, through the involvement of multiple analysts as well as soliciting views from three groups of participants, thereby constituting triangulation [19, 20]. Dependability was achieved through ensuring that the analysis was grounded in rich data, as illustrated by verbatim quotes. A clear exposition of study participants and thick descriptions of the background, methods, and findings helped in achieving transferability. Doing so was a way of placing the data in its proper context to allow the reader to understand participants’ views in relation to the given topic. It was assumed that providing the nuances of the contexts in which discussions were held would help the reader to better understand the interview excerpts. Furthermore, use of a purposive sampling strategy also contributed to transferability. Participants comprising the study sample were relevant to the topic as they had experienced care in child-friendly spaces. Confirmability which often seeks to mitigate researcher bias was ensured through pretesting of the data collection tool and keeping an audit trail of the entire research process.

### Ethical considerations

Ethical clearance for this study was obtained from the University of KwaZulu-Natal’s Biomedical Research Ethics Committee (BREC) (Approval BE 298/18) and the Directorate for Health Research and knowledge management (KZ_201809_011). Gatekeeper permission was obtained from the HIV, AIDS and STIs Directorate at the KwaZulu-Natal Department of Health Provincial Office and the Zoe-life Directorate, respectively. We sought written informed consent from every participant prior to conducting the respective interviews. We also sought assent from all the children that participated in the study using a child-friendly story-based information sheet and assent form.

## Results

### Demographic characteristics of children and PCGs and KidzAlive trained HCWs

KidzAlive trained HCWs  participating in this study comprised eight nurses and 20 HIV counsellors, with ages and experience working with HIV seropositive children ranging from 25-46 years 3–12 years, respectively (see Table 1). The child-PCG pairs were mostly from low and middle-income families. Most (37%) of the children were aged 7–8 years, with females and males comprising 57 and 43%, respectively. All the children were both school-going and on ART, with 30 and 17% being fully and partially disclosed to, respectively. As high as 53% of the children were not aware of their HIV seropositive status. Of those that were either partially or fully disclosed to, 71% were disclosed to when they were aged between 6 and 8 years, while 21% knew about their HIV seropositive status at ages 9–12 years, and 7% between the ages 2–5 years (Table 2). Most PCGs interviewed were female (90%), 63% were the biological mother to the child, 83% had received a minimum of high school education and 50% were HIV seropositive, while 30% were negative and 20% did not know their HIV status (Table 2).

**Table 1:** Demographic characteristics of KidzAlive trained and mentored HCWs

Variable	Frequency	%	Variable
Sex	Male	1	5%
	Female	19	95%
Age (years)	18-25	3	15%
	26-30	4	20%
	31-40	8	40%
	40 and upwards	5	25%
Professional designation	Professional nurse	8	40%
	HIV/AIDS Counsellor	12	60%
Number of years working with children	1-3	9	45%
	4-6	2	10%
	7-9	7	35%
	10 years and above	2	10%

**Table 2:** Demographic characteristics of children and primary caregivers

Variable	Frequency	%	Variable
Sex	Male	3	10%
	Female	27	90%
Age of primary caregiver	18–25	8	27%
	26–30	10	33%
	31–40	9	30%
	40 and upwards	3	10%
Marital status	Married/cohabiting	19	63%
	Single	7	23%
	Divorce/separated /widowed	4	13%
Relationship to the child	Biological mother	19	63%
	Biological father	1	3%
	Grand parent	2	7%
	Uncle/Aunt	4	13%
	Sibling	4	13%
	Other	1	3%
Level of education of primary caregiver	No formal education	2	7%
	Up to primary school	3	10%
	Up to high school and beyond	25	83%
HIV status of primary caregiver	HIV positive	15	50%
	HIV negative	9	30%
	Unknown	6	20%
Child’s age	5–6 years	4	13%
	7–8 Years	11	37%
	9 to 10 years	6	20%
	11–12 years	9	30%
Child’s sex	Male	13	43%
	Female	17	57%
Child’s age of diagnosis	0–2 years	12	40%
	3–4 years	9	30%
	5–6 years	3	10%
	7–8 Years	3	10%
	9 to 10 years	2	7%
	11–12 years	1	3%
Disclosure status of child	Not disclosed	16	53%
	Partially disclosed	5	17%
	Full disclosure	9	30%
Child age of disclosure	2–5 years	1	7%
	6–8 years	10	71%
	9–12 years	3	21%

### Themes and sub-themes

Five themes were identified from the analysis, namely: (1) Child participation and involvement in their own healthcare journey; (2) HCW confidence in the provision of child-friendly HIV services; (3) Transformation of the PHC setting into a therapeutic environment for children; (4) PCGs’ participation in the care of their children; and (5) Barriers to the effective use of child-friendly spaces in healthcare settings. Five sub-themes emerged from the analysis and these are: (1) Space challenges; (2) Clashing PHC facility priorities and poor management support; (3) Inadequate training of how to maximise the child-friendly space; (4) The need for continued replenishment of the child-friendly spaces; and (5) Existing child-friendly spaces inappropriate for much older children.

#### Child participation and involvement in their own healthcare journey

KidzAlive trained and mentored HCWs and PCGs perceived the use of child-friendly spaces to be an effective strategy for reducing the child’s apprehension towards the HCW providing care, the healthcare facility and clinical procedures.

*AQ1: “When children come to the health facility, they fear the nurses or fear being pricked by the nurse either during HIV testing or during blood draw for viral load analysis. However, with the child-friendly spaces, the same children have completely forgotten about all that pain and anxiety such that when they come into the counselling room, they would be itching to sit in the child-friendly space either to draw, colour-in or listen to the story of Sibusiso”* 33-year-old nurse, uMkhanyakude District.

*AQ2: “I enjoyed playing in the child-friendly spaces. I liked the story of Sibusiso and Bheki. Before, when I used to come to the health facility, the nurse only asked me if I was feeling ok. After that, she would speak to my mom”*, 6-year-old girl, eThekwini District.

Children reported that they felt less fearful of being given an injection or of the KidzAlive trained HCWs drawing blood for viral load assessment.

*AQ3: “When the nurse wants to prick me, she says that I must be brave like Sibusiso. When she finishes, I can go and play with my toys in the play area. I enjoy playing in this area because I previously used to just sit on the bench”,* 6-year-old boy, Zululand District.

KidzAlive trained HCWs and PCGs reported that children had a renewed interest in their care and accessing the PHC facility. The children were also comfortable and confident to interact with KidzAlive trained HCWs. This was evidenced by the increased number of questions they asked during clinic visits. Children’s openness to communicate their feelings, fears and concerns about using antiretroviral therapy (ART), playing with toys and storytelling were observed as marked improvements.

*AQ4: “I now enjoy going to the health facility because we learn a lot about germs and how to put them to sleep using goodnight medicine. I like my nurse because she makes me laugh and we play games, and sometimes she tells me a story. I also ask her questions when I don’t understand and she always asks me how I am feeling”,* 5-year-old boy, eThekwini District.

PCGs also welcomed the child-friendly spaces, citing that they enabled the KidzAlive trained and mentored HCWs to spend more time talking to their children and helping them with the different aspects of HIV service.

*AQ6: “Now children can talk to the healthcare worker when they are in the child-friendly spaces because they have time to talk to our children. The children are always asking us questions about why they are at the health centre. Thus, the healthcare worker answers and gives the child adequate information to make them understand more about HIV and why they should take medicine to make soldier cells strong”,* 40-year-old father to a 10-year-old boy, uMgungundlovu District.

Due to the play therapy tools including storytelling using toys and drawings, KidzAlive trained HCWs recounted instances when children disclosed other issues apart from HIV such as abuse, which allowed the KidzAlive trained HCWs to provide a more informed service.

*AQ7: “I think he enjoys visiting the clinic compared to the previous visits where he would be on my lap for the duration of the visit”,* 36-year-old primary caregiver to a 7-year-old boy, eThekwini District.

#### HCW confidence in the provision of child-friendly HIV services

KidzAlive trained HCWs, children and their PCGs were in agreement that child-friendly spaces had improved communication among children, KidzAlive trained HCWs and PCGs. This improvement was attributed to the active use of play therapy techniques such as games, drawing and colouring, in which both the children and their caregivers participated. KidzAlive trained HCWs often gave an example of the ‘hand of safety’, a colour-in art template on which the child was asked to colour in and write the names of people that were important in their lives on each finger.

BQ1: *“The child-friendly spaces and activities conducted in the space are user-friendly and they suit the ages of our children. You know that all children enjoy playing with toys, drawing and colouring with crayons so this was a very clever way to make our children lose their fear for nurses. Even the children now know about the stigma because of the ‘hand of safety’ drawing which is used to identify the people that support the child at home, thereby providing an opportunity for the the KidzAlive trained HCW to address stigma. My grandchild really loves their nurses because of these spaces”,* 60-year-old, grandmother to a 7-year-old boy, eThekwini District.

In addition, KidzAlive trained HCWs also used age-appropriate methods of relaying information in the form of storytelling and metaphors to communicate important health care messages to children.

HCWs also reported that by using the colour-in drawings, they were able to understand the people forming the child’s circle of support.

*BQ3: “Visuals make it easy to share a difficult subject with the child. Crayon/colouring-in makes the child feel more at ease to talk and interact with the caregiver. The hand of safety tool also assures the primary caregiver that the child will not share information with anyone outside the hand of safety”,* 25-year-old nurse, eThekwini District.

Some KidzAlive trained HCWs reported that they allowed the children to display their completed artwork on the walls of the child-friendly space to increase children’s ownership of the space. Further, they indicated that displaying the artwork encouraged re-use on subsequent visits to provide a conversation starter with the child, provide an effective recap and promote further engagement and continuity.

*BQ4: “When children draw their artwork, we stick it on the wall for them to see it and if it’s not finished, we keep it and give it back to the child. This increases their willingness to come back and finish their drawings and it is an icebreaker for us because we are sometimes intimidated by our fear of talking to children and not knowing how to start the conversation with them. It is also easy to provide a recap using their artwork”,* 32-year-old HIV counsellor, Zululand District.

KidzAlive trained HCWs and PCGs agreed that the toys and other play paraphernalia available in the child-friendly space created a diversion for the children, thereby putting them at ease and reducing their anxiety. Children shared similar sentiments. 

*BQ5: “When I am in the child-friendly space, I feel like I am at my school, but it’s more fun because I get to do artwork and the nurse tells me funny stories, which make me laugh. I really enjoy playing in this space, and I don’t mind coming to the clinic. Before, I would just sit on the bench, so it was boring to come to the clinic with my mother”,* 6-year-old boy, eThekwini District.

#### PCGs’ participation in the care of their children

PCGs reported that KidzAlive trained HCWs taught them how to communicate with their children through play. They indicated that through child-friendly spaces, disclosing the child’s HIV seropositive status was made easier.

*CQ1: “Before, I feared to bring my grandchild to the clinic. But when I went to the clinic and spoke to the nurse and they saw the place where they would provide the services to my grandson, my worry was lessened. I have disclosed to my child that they have HIV in the child-friendly space, with the support of the nurse who answered all the questions using a language that the child understood. I must confess that disclosure has made my child understand the reason why they are taking meds and it has also freed me as a parent and I now have hope that my child will grow and be a success in life”,* 55-year- old grandmother to an 8-year-old boy, uMkhanyakude District.

The KidzAlive trained HCWs were also reported to have given the PCGs an opportunity to explore the use of play therapy skills, especially during the disclosure process. PCGs were appreciative of this new knowledge and expertise. They felt empowered to continue using play therapy in the home setting to teach their children and communicate important healthcare messages.

*CQ2: “The experience was painful yet educational because it taught me that it is important to tell my child about their illness, so they can accept it in their early stages of life. The health provider taught me how to talk to my child and how to play games with her, which has improved our relationship. I am no longer afraid because previously, the child would ask me questions which were very hard to answer. I like our sessions at the clinic because now the children are more recognised, and the service is directly focused on them”,* 34-year-old mother to a 9-year-old girl, Zululand District.

#### Transformation of the PHC setting into a therapeutic environment for children

Children reported that the child-friendly spaces transformed the PHC setting into a welcoming environment, something that was previously lacking. They especially liked the colourful ambience of the child-friendly spaces which created familiarity and cheered them up during their PHC visits.

*DQ1: “The child-friendly spaces have provided us with a space of our own, where we are accepted as children and we do children’s activities. The spaces are very colourful, and they remind us of our school environment which is very nice because it makes us forget that we are here for HIV. This reduces our fear and discomfort”,* 9-year-old girl, eThekwini District.

PCGs concurred with their children, highlighting that the child-friendly space had transformed the healthcare environment from being a place for adults, to a place where children feel at ease.

*DQ3: “Before, the health facility never used to care about changing the place to accommodate the needs of both children and adults. Now the children are well accommodated and there are books for them, toys and a place where they can play. This is important for our children who are HIV positive and must visit the clinic every month. These spaces have really changed the services for children”,* 38-year-old father to a 7-year-old boy, Zululand District.

PCGs also highlighted that the presence of the child-friendly space had increased their willingness to bring children to healthcare facilities adding that these child-friendly spaces were a visible symbol of the improved quality of care available for their children at the health facility.

*DQ4: “When you walk into the health facility and you see the child-friendly space, you see that the clinic environment has changed, and it is very accommodating for children. This is very pleasing to us as parents to see that the clinic makes an effort for our children”,* 55-year-old grandfather to a 6-year-old boy, eThekwini District.

Most KidzAlive trained HCWs reported that the provision of child-friendly spaces in PHC facilities was a shift from the usual care process because such spaces were historically offered only in private clinics and hospitals. The introduction of this approach was appreciated by the participants due to its sensitivity to the needs of their children during visits to local PHC facilities.

*DQ5: “I had only seen children’s play areas at private clinics and private hospitals. Now even those of us in the community have a private hospital experience at our health facilities because of KidzAlive. The community is commenting when they see the child-friendly space, telling us that they like the way we are trying to include their children”,* 42-year-old HIV counsellor, eThekwini District.

PCGs added that they had shared their positive experiences of accessing services in the child-friendly spaces with other PCGs and family members. Similarly, children also reported that they had shared their experiences with other children.

#### Barriers to the effective use of child-friendly spaces in healthcare settings

##### Challenges in finding space fit for purpose

KidzAlive trained HCWs reported that space for establishing child-friendly spaces was scarce and they had to create temporary spaces.

*EQ1: “There simply is no space to create child-friendly spaces. We sometimes create temporary spaces. Well, this works for us, at least for now”,* 40-year-old HIV Counsellor, Zululand District.

In addition, KidzAlive trained HCWs reported that the Ideal Community Policy forbids sticking posters onto walls, making it difficult for them to decorate walls with colourful posters to make them more appealing to children.

Several PCGs had concerns regarding confidentiality and the fear of being labelled (stigma) by community members. They indicated that the child-friendly space was known to be a place where children living with HIV received care and support, thus inadvertently promoting stigmatisation.

*EQ3: “My fear is that the community now knows that children that go to the child-friendly spaces are HIV positive. I don’t want people to know my business so sometimes I think the space can make me disclose unwillingly that I am positive and that I infected my child”,* 34-year-old mother to a 9-year-old girl, Zululand District*.*

##### Health facility priorities and management support

The child-friendly space was not high on the PHC facility manager’s list of priorities. This was evidenced by constantly changing the child-friendly room, thereby causing confusion in terms of its location. To prevent this, the KidzAlive trained HCWs recommended the training and orientation of all facility staff to increase their understanding of the value of the child-friendly space.

*EQ4: “My manager doesn’t understand the programme and when I am using the tools, more time is used, and she complains that the health facility gets congested. I must explain that providing services to children takes longer as I must counsel both parties [primary caregiver and the child] and ensure that they understand what I would be talking about. I think its best that the managers also receive training so that they understand the whole process and what it takes”,* 32-year-old HIV counsellor, Zululand District*.*

##### Inadequate training of how to maximise the child-friendly space

KidzAlive trained HCWs reported that they needed additional training on how to fully utilise the child-friendly space.

*EQ5“Although we have created the child-friendly spaces, we still need additional training and support on how to fully utilise them to improve our services to children and their caregivers”,* 25-year-old HIV counsellor, uMkhanyakude District*.*

The participants also added that they needed more training on how to create adolescent friendly facilities for older children and adolescents to complement the existing KidzAlive training.

*EQ6: “We still need training on how to create adolescent friendly chill-rooms for older children and adolescents because the spaces we have are for children. Dealing with adolescents is not easy so we need training on how to engage and keep them interested”,* 32-year-old, HIV counsellor, Zululand District*.*

However, KidzAlive trained HCWs highlighted that the concept of child-friendly spaces complements the existing adolescent chill rooms currently being implemented through the adolescent-friendly initiative by South Africa’s National Department of Health.

*EQ7“The Department of Health has asked us to put in place a chill-room for older children and adolescents to increase their willingness to visit the health facility and participation in their care. I am happy to say that the KidzAlive intervention provides that continuity of care for children. After they outgrow the child-friendly spaces, they can visit the adolescent chill rooms. So, the child-friendly spaces were a very good idea for younger children”,* 35-year-old nurse, uMgungundlovu District.

##### The need for continued replenishment of the child-friendly spaces

KidzAlive trained HCWs reported that children were taking toys and other related paraphernalia from the child-friendly spaces and these were not being replenished to avoid depletion.

*EQ8: “Children take toys from the child-friendly space and when you try to take the toy back after the session, the child refuses and there is a lot of crying. In the end, we allow them to take the toys away and we never see them again. I think we need to start getting some balloons as giveaways. Importantly, because we lose so many toys, we need to replace them and for that, we need the support from our facility manager”,* 46-year-old nurse, uMkhanyakude District.

##### Existing child-friendly spaces not appropriate for much older children

Older children (9–12 years) were concerned that existing furniture, games and activities were inappropriate for them. They requested for KidzAlive to adopt an adolescent-friendly approach when developing interventions for older children.

*EQ9: “I can’t sit in those tiny pre-school chairs because I’m big. I would look ridiculous. Hence, I think we as older children need some space of our own. Some of the games that are played in these spaces are for much younger children. Maybe something for us older children will also improve our experiences of care. But I must say that healthcare workers are very attentive, and they now ask a lot of questions and allow me to ask questions. They provide simple answers which are easy to understand”,* 11-year-old girl, uMkhanyakude District.

## Discussion

The findings suggest that child-friendly spaces contribute to the centredness of care for children in PHCs. This was evidenced by the increased involvement and participation of children, increased PCGs participation in the care of their children and the positive transformation of the PHC to a therapeutic environment for children. Several barriers impeding the success of child-friendly spaces were reported, including space constraints; clashing health facility priorities; inadequate management support; inadequate training on how to maximise the child-friendly spaces, and their inappropriateness for older children.

The existence of child-friendly spaces has created a common ground for HCW-child-PCG engagement. Child-friendly spaces have also helped in creating a conducive environment for discussing issues to do with disclosure due to its sensitivity to the use of age-appropriate activities that involve children, their PCGs and HCWs. Consistent with a study conducted in Nigeria, the creation of a child-friendly clinic yielded positive health outcomes among children including adherence to medication, retention in care, a reduced loss to follow-up in children and improved care experiences [22].

Child-friendly spaces have also contributed to the creation of a learning environment for children where they learn through play using play paraphernalia that is familiar, to which they can relate. This has contributed to their increased understanding of HIV and reduced their fear of HCWs and the PHC facility in general, by providing a pleasant diversion. Play therapy tools and techniques used in these child-friendly spaces, such as storytelling, play dough, art and puppets were perceived to be complementary to the child-friendly space. These play therapy techniques were perceived to be effective, especially during the provision of HIV services to younger children. Such techniques helped to prepare children for painful procedures such as pricking during HIV testing and needle injections when drawing blood for viral load assessments. Similar findings were reported in several studies reporting the outcomes of providing children with pre-operative care in child-friendly environments where children and their PCGs experienced reduced anxiety. Medical procedures often trigger anxiety [63–67].

The findings also suggest that child-friendly spaces have a positive transformative effect on children and PCGs' perception of the quality of care at the PHC facility. Similar findings were reported in Nigeria where the child-friendly clinic had increased healthcare seeking for children living with HIV [22].

The child-friendly space was perceived to be an age-appropriate innovation in HIV care for children, as they create a safe space for children to express themselves, share their experiences and be involved in their own care. This resonates with the general philosophy of a rights-based approach [14]. Consistent with the findings of the studies conducted in Sri Lanka, Uganda, Yemen, the child-friendly space approach has made it easier for the KidzAlive trained HCWs to provide a more informed service and subsequently refer children to other service providers [39, 43].

Children highlighted that the colourful environment provided through child-friendly spaces appealed to them, which made them forget why they were at the health facility. This is in line with findings from several studies reporting the importance of aesthetics in healthcare spaces for children [68]. Particularly outstanding is a study assessing children’s perceptions of the paediatric ward in Malaysia which reported that bright colours were perceived to create a cheerful ambience in the paediatric ward. This improved the patients’ mood, lessened anxiety and depression [64, 68].

While the experiences of all the beneficiaries of the child-friendly spaces were generally positive, non-versatility of the existing concept to suit older children above 10 years was an important challenge. However, this challenge may be minimised. For example, the South African National Department of Health can accelerate the creation of adolescent-friendly spaces through its National Adolescent-Friendly Clinics Initiative (NAFCI) to cater for older children [69, 70]. Doing so will ensure continuity of care from childhood to adolescence in PHC settings in South Africa.

In addition, limited support of the concept of child-friendly spaces by facility management requires attention. Suggestions made by KidzAlive trained HCWs regarding the training of PHC facility management on child-friendly approaches are worth noting. Such trainings are improtant because they provide opportunities for promoting stakeholder buy-in. Several studies suggest that PHC facility management training before initiating innovative healthcare programmes is beneficial to their success as it increases their buy-in and support [71–75].

Lastly, the current PHC policy inhibits the creation of child-friendly spaces, particularly the existing Ideal Community Policy [76] that prohibits sticking of any posters or drawings on the facility’s walls. This makes decorating of the child-friendly room or child-friendly space difficult. It also makes it difficult for children to showcase their artistic masterpieces drawn during sessions, yet these are important to fostering their ownership of the space.

### Implications of the study

This study provided the much-needed evidence from healthcare users and healthcare providers pertaining to the implementation of child-friendly spaces and how they have influenced healthcare provision for children. It contributes to building the corpus of evidence on child-friendly approaches to HIV care for children. The study also provides a reflective opportunity to PHC managers about child-friendly spaces and their potential contribution for possible scale-up.

### Limitations

The major limitation of KidzAlive is that that study does not address the socio-demographic background of the children and their PCGs under KidzAlive, which could have given readers more insight into their responses and experiences. An additional limitation is the fact that we did not interview health facility managers and other district managers who could have provided valuable information which could improve the intervention. Furthermore, we neither returned the transcripts to participants for comment and/or correction nor did we share our findings with the participants for them to provide feedback. These two critical steps in qualitative research were omitted due to funding constraints and challenges with accessing the child-PCG pairs. Lastly, due to the qualitative nature of the study, we cannot generalise the findings to other facilities where child-friendly spaces have been created. However, the study served its purpose of providing insight into user-provider experiences of the new concept of child-friendly spaces in PHC settings in KwaZulu-Natal, South Africa. The findings also demonstrate the acceptability and utility of child-friendly spaces intervention in PHC facilities. This study paves way for complementary rigorous studies that can determine the impact of these child-friendly spaces on children’s health outcomes.

## Conclusion

The child-friendly spaces intervention complements the adolescent-friendly initiatives currently being implemented by the National Department of Health of South Africa. It also contributes to the knowledge of how child-centred care approaches such as child-friendly spaces can contribute to the delivery of age-appropriate and client-centred HIV care for children aged 5–12 years, especially in resource constrained settings.

## Data Availability

Qualitative interview transcripts and the corresponding NVivo files are only visible to the direct research team and are not publicly available in accordance with permissions stated in the Ethics Approval from the KwaZulu-Natal Biomedical Ethics Committee (BREC).

## Abbreviations

HCWs: Healthcare Workers
IOM: Institute of medicine
PCGs: Primary caregivers
PHC: Primary healthcare Clinics
RA: Research Assistants
UNCRC: United Nations Convention on the Rights of the Child
UNICEF: United Nations Children’s Fund

## References

[1] Domek GJ (2010). Debunking common barriers to pediatric HIV disclosure. J Trop Pediatr.

[2] The LCAHJTLC, health a (2018). Children and youth are crucial in the global AIDS response. Lancet Child Adolesc Health.

[3] Coyne I, O’Mathúna DP, Gibson F, Shields L, Leclercq E, Sheaf G (2016). Interventions for promoting participation in shared decision-making for children with cancer. Cochrane Database Syst Rev.

[4] Oliveras C, Cluver L, Bernays S, Armstrong A (2018). Nothing about us without RIGHTS—meaningful engagement of children and youth: from research prioritization to clinical trials, implementation science, and policy. J Acquir Immune Defic Syndr.

[5] Mark D, Geng E, Vorkoper S, Essajee S, Bloch K, Willis N (2018). Making implementation science work for children and adolescents living with HIV. J Acquir Immune Defic Syndr.

[6] Ford K, Dickinson A, Water T, Campbell S, Bray L, Carter B (2018). Child centred care: challenging assumptions and repositioning children and young people. J Pediatr Nurs.

[7] MacKean GL, Thurston WE, Scott CM (2005). Bridging the divide between families and health professionals’ perspectives on family-centred care. Health Expect.

[8] Baker AJB (2001). Crossing the quality chasm: a new health system for the 21st century. BMJ.

[9] Carter B, Bray L, Dickinson A, Edwards M, Ford K (2014). Child-centred nursing: promoting critical thinking: sage.

[10] Kilkelly U, Savage E (2013). Child-friendly healthcare.

[11] Donnelly M, Kilkelly U (2011). Child-friendly healthcare: delivering on the right to be heard. Med Law Rev.

[12] Coyne I, Holmström I, Söderbäck M (2018). Centeredness in healthcare: a concept synthesis of family-centered care, person-centered care and child-centered care. J Pediatr Nurs.

[13] Jo Delaney L (2018). Patient-centred care as an approach to improving health care in Australia. Collegian.

[14] Ford K, Campbell S, Carter B, Earwaker L (2018). The concept of child-centered care in healthcare: a scoping review protocol. JBI Database System Rev Implement Rep.

[15] Foster M (2015). A new model: the family and child centered care model. Nursing Praxis New Zealand.

[16] Birch J, Curtis P, James A (2007). Sense and sensibilities: in search of the child-friendly hospital. Built Environ.

[17] Taylor B (2006). Giving children and parents a voice-the parents’ perspective. Paediatr Nurs.

[18] Majamanda MD, Munkhondya TEM, Simbota M, Chikalipo M (2015). Family centered care versus child centered care: the malawi context. Health.

[19] Burrows L, Wright JJS, Education, Society (2004). The good life: New Zealand children’s perspectives on health and self. Sport Educ Soc.

[20] Mutambo C, Hlongwana KJ (2019). Healthcare Workers’ Perspectives on the Barriers to Providing HIV Services to Children in Sub-Saharan Africa. AIDS Res Treat.

[21] Gyamfi E, Okyere P, Appiah-Brempong E, Adjei RO, Mensah KA (2015). Benefits of disclosure of HIV status to infected children and adolescents: perceptions of caregivers and health care providers. J Assoc Nurses AIDS Care.

[22] Ugwu OR (2017). Impact of a child-friendly clinic on retention of HIV-infected children in care: an intervention study. Nigerian J Paediatr.

[23] Putta N, Lovich R, Kean S, Mark D (2018). A child-centered approach for HIV programs.

[24] O’Malley G, Beima-Sofie K, Feris L, Shepard-Perry M, Hamunime N, John-Stewart G (2015). “If I take my medicine, I will be strong:” Evaluation of a pediatric HIV disclosure intervention in Namibia. J Assoc Nurses AIDS Care.

[25] Brandt L, Beima-Sofie K, Hamunime N, Shepard M, Ferris L, Ingo P (2015). Growing-up just like everyone else: key components of a successful pediatric HIV disclosure intervention in Namibia. AIDS.

[26] Health SANDo. Disclosure guidelines for children and adolescents in the context of HIV, TB and non-communicable diseases. http://www.health.gov.za/index.php/hiv-aids-tb-and-maternal-and-child-health?download=1696:hiv-disclosure-guideline-for-children-and-adolescent-2016-1.

[27] Organization WH (2011). Guideline on HIV disclosure counselling for children up to 12 years of age.

[28] Gyamfi E, Okyere P, Enoch A, Appiah-Brempong E, Rights H (2017). Prevalence of, and barriers to the disclosure of HIV status to infected children and adolescents in a district of Ghana. BMC Int Health Hum Rights.

[29] Oti SO (2013). HIV and noncommunicable diseases: a case for health system building. Curr Opin HIV AIDS.

[30] Pasricha A, Deinstadt RTM, Moher D, Killoran A, Rourke SB, Kendall CE (2013). Chronic care model decision support and clinical information systems interventions for people living with HIV: a systematic review. J Gen Intern Med.

[31] Lambert V, Coad J, Hicks P, Glacken M (2014). Young children’s perspectives of ideal physical design features for hospital-built environments. J Child Health Care.

[32] Creswell JWJL, Publications TOS (2009). Research design: qualitative and mixed methods approaches.

[33] Hamdan AB, AlShammary S, Tamani JC, Peethambaran S, Hussein M, AlHarbi M. The impact of creating a child-friendly hospital environment in pediatric cancer patients and their families in comprehensive cancer center at King Fahad Medical city. Curr Pediatr Res. 2016;20.http://www.currentpediatrics.com/articles/articles/the-impact-of-creating-a-childfriendly-hospital-environment-in-pediatric-cancer-patients-and-their-families-in-comprehensive-cance.html.

[34] Weber FS (2010). The influence of playful activities on children’s anxiety during the preoperative period at the outpatient surgical center. J Pediatr (Rio J).

[35] McCarthy EA, Subramaniam HL, Prust ML, Prescott MR, Mpasela F, Mwango A (2017). Quality improvement intervention to increase adherence to ART prescription policy at HIV treatment clinics in Lusaka, Zambia: A cluster randomized trial. PloS one.

[36] ISTCA (2008). Child friendly spaces in emergencies: a handbook for save the children staff. Save the Children.

[37] Metzler J, Atrooshi A, Khudeda E, Ali D, Ager A. Evaluation of child friendly spaces. In: Iraq field study report: a MoLSA-implemented CFS in Domiz Refugee Camp: World Vision International, Columbia University Mailman School of Public Health and UNICEF; 2014. https://www.researchgate.net/profile/Janna_Metzler/publication/272815902_Iraq_SC_CFS_Evaluation_Field_Study_Report/links/54ef42840cf2432ba6563830.pdf.

[38] UNICEF (2011). Principles for child friendly spaces in emergencies.

[39] WVI. Child friendly spaces: a structured review of the current evidence-base: World Vision International; 2012.

[40] Davis K, Iltus S (2009). A practical guide for developing child friendly spaces.

[41] Kostelny K, Wessells M. Child-friendly spaces: Promoting children’s resiliency amidst war. In Handbook of resilience in children of war. New York: Springer; pp. 119–129.

[42] Madfis J, Martyris D, Triplehorn C (2010). Emergency safe spaces in Haiti and the Solomon Islands. Disasters.

[43] Sabina N. Report of emergency response evalutation and lessons learned of response in Satkhira flood 2011: Sunderban Sub cluster on child protection in emergencies; 2012. https://reliefweb.int/sites/reliefweb.int/files/resources/CFS_Literature_Review_final_Aug_2012.pdf.

[44] SADoH. Psychosocial support for children and adolescents infected and affected by HIV and AIDS. South African Department of Health. http://www.health.gov.za/index.php/shortcodes/2015-03-29-10-42-47/2015-04-30-08-18-10/2015-04-30-08-25-54?download=594:psychological-support-pss-for-children-and-adolescents-infected-and-affected-by-hiv-and-aids.

[45] Wong V, Macleod I, Gilks C, Higgins D, Crowley S (2006). The lost children of universal access – issues in scaling-up HIV testing and counselling. Vulnerable Children Youth Stud.

[46] Rwemisisi J, Wolff B, Coutinho A, Grosskurth H, Whitworth J (2008). ‘What if they ask how I got it?’ Dilemmas of disclosing parental HIV status and testing children for HIV in Uganda. Health Policy Plan.

[47] Mkwanazi N, Rochat T, Coetzee B, Bland R (2013). Mothers’ and health workers’ perceptions of participation in a child-friendly health initiative in rural South Africa. Health.

[48] Bandura A (1977). Social learning theory. Englewood cliffs: Prentice Hall.

[49] Piaget J (1976). Piaget’s theory. Piaget and his school.

[50] Holzman L. Vygotsky at work and play: Routledge; 2016.

[51] O’Neill S, Rajendran K, Halperin JM (2012). More than child’s play: the potential benefits of play-based interventions for young children with ADHD. Expert Rev Neurother.

[52] Bratton SC, Ray D, Rhine T, Jones L (2005). The efficacy of play therapy with children: a meta-analytic review of treatment outcomes. Prof Psychol Res Pract.

[53] Landreth G (2002). Play therapy: the art of the relationship.

[54] Schultz W (2016). Child-centered play therapy. Reason Papers.

[55] Crenshaw DA, Kenney-Noziska S (2014). Therapeutic presence in play therapy. Int J Play Therapy.

[56] Organization WH (2015). United States President’s Emergency Plan for AIDS Relief (PEPFAR), Joint United Nations Programme on HIV/AIDS (UNAIDS) and the Global Fund to Fight AIDS, Tuberculosis and Malaria. A guide to monitoring and evaluation for collaborative TB/HIV activities.

[57] Health NDo. Adherence guidelines for HIV, TB and NCDs: policy and service delivery guidelines for linkage to care, adherence to treatment and retention in care. Pretoria; National Department of Health 2016.

[58] Health SANDo (2016). Disclosure guidelines for children and adolescents in the context of HIV, TB and non-communicable diseases.

[59] Parker I (2013). Discourse analysis: dimensions of critique in psychology. Qual Res Psychol.

[60] Tong A, Sainsbury P, Craig J (2007). Consolidated criteria for reporting qualitative research (COREQ): a 32-item checklist for interviews and focus groups. Int J Qual Health Care.

[61] Reddy P, Meyer-Weitz A, Van den Borne B, Kok G, Weijts W (1998). The learning curve: health education in STI clinics in South Africa. Soc Sci Med.

[62] Squires A (2009). Methodological challenges in cross-language qualitative research: a research review. Int J Nurs Stud.

[63] William Li HC, Lopez V, Lee TL (2007). Effects of preoperative therapeutic play on outcomes of school-age children undergoing day surgery. Res Nurs Health.

[64] Lambert V, Coad J, Hicks P, Glacken M (2014). Social spaces for young children in hospital. Child Care Health Dev.

[65] Rice G, Ingram J, Mizan J (2008). Enhancing a primary care environment: a case study of effects on patients and staff in a single general practice. Br J Gen Pract.

[66] Lerwick JL (2016). Minimizing pediatric healthcare-induced anxiety and trauma. World J Clin Pediatr.

[67] da Silva RDM, Austregésilo SC, Ithamar L, Lima LS (2017). Therapeutic play to prepare children for invasive procedures: a systematic review. J Pediatr (Rio J).

[68] Ghazali R, Abbas MY, Jalalkamali N (2013). Healing environment in paediatric wards: from research to practice. Procedia Soc Behav Sci.

[69] Kuo C, LoVette A, Pellowski J, Harrison A, Mathews C, Operario D (2019). Resilience and psychosocial outcomes among south African adolescents affected by HIV. Aids.

[70] Joanna Briggs Institute Critical Appraisal (2017). Critical Appraisal Tools.

[71] Doherty J, Gilson L, Shung-King M (2018). Achievements and challenges in developing health leadership in South Africa: the experience of the Oliver Tambo Fellowship Programme 2008–2014. Health Policy Planning.

[72] Sonnino RE (2016). Health care leadership development and training: progress and pitfalls. J Healthc Leadersh.

[73] Matovu JKB, Wanyenze RK, Mawemuko S, Wamuyu-Maina G, Bazeyo W, Olico O (2011). Building capacity for HIV/AIDS program leadership and management in Uganda through mentored Fellowships. Glob Health Action.

[74] Sebuliba I, Lindan C, Baryamutuma R, Kyomugisha C, Muhumuza S, Bazeyo W, et al. Improving the ability of districts in Uganda to monitor their HIV programs. East Afr J Appl Health Monitor Eval. 2018;2018(2) http://eajahme.com/wp-content/uploads/2018/03/Sebuliba_FINAL.pdf. PMC6433397 30918924

[75] Jones DS, Tshimanga M, Woelk G, Nsubuga P, Sunderland NL, Hader SL (2009). Increasing leadership capacity for HIV/AIDS programmes by strengthening public health epidemiology and management training in Zimbabwe. Hum Resour Health.

[76] Hunter JR, Chandran TM, Asmall S, Tucker J-M, Ravhengani NM, Mokgalagadi Y (2017). The Ideal Clinic in South Africa: progress and challenges in implementation. South African Health Rev.

